# Novel aspects of ethylene glycol catabolism

**DOI:** 10.1007/s00253-024-13179-2

**Published:** 2024-06-11

**Authors:** Tetsu Shimizu, Masayuki Inui

**Affiliations:** 1 Research Institute of Innovative Technology for the Earth, 9-2, Kizugawadai, Kizugawa-shi, Kyoto, 619-0292 Japan; 2 Division of Biological Science, Graduate School of Science and Technology, Nara Institute of Science and Technology, 8916-5 Takayama, Ikoma, 630-0192 Japan

**Keywords:** Ethylene glycol, PET, Sustainable process, Glycerate pathway, Β-Hydroxyaspartate cycle

## Abstract

**Abstract:**

Ethylene glycol (EG) is an industrially important two-carbon diol used as a solvent, antifreeze agent, and building block of polymers such as poly(ethylene terephthalate) (PET). Recently, the use of EG as a starting material for the production of bio-fuels or bio-chemicals is gaining attention as a sustainable process since EG can be derived from materials not competing with human food stocks including CO_2_, syngas, lignocellulolytic biomass, and PET waste. In order to design and construct microbial process for the conversion of EG to value-added chemicals, microbes capable of catabolizing EG such as *Escherichia coli*, *Pseudomonas putida*, *Rhodococcus jostii*, *Ideonella sakaiensis*, *Paracoccus denitrificans*, and *Acetobacterium woodii* are candidates of chassis for the construction of synthetic pathways. In this mini-review, we describe EG catabolic pathways and catabolic enzymes in these microbes, and further review recent advances in microbial conversion of EG to value-added chemicals by means of metabolic engineering.

**Key points:**

• *Ethylene glycol is a potential next-generation feedstock for sustainable industry.*

• *Microbial conversion of ethylene glycol to value-added chemicals is gaining attention.*

• *Ethylene glycol-utilizing microbes are useful as chassis for synthetic pathways.*

## Introduction

**Fig. 1:**
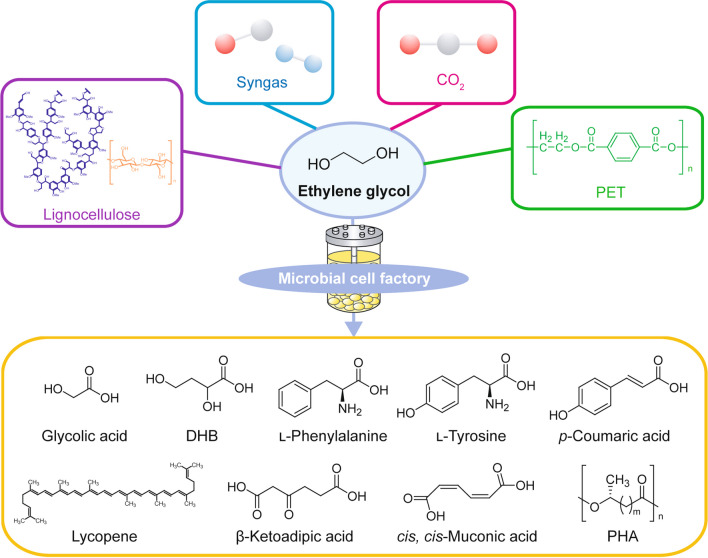
Microbial cell factories for the conversion of ethylene glycol to value-added chemicals

Ethylene glycol (EG) is an industrially important two-carbon diol used as a solvent, antifreeze agent, and building block of polymers such as poly(ethylene terephthalate) (PET). Currently, EG is predominantly produced from fossil fuels by hydration of ethylene oxide, while a small amount of EG is produced from renewable resources by dehydration of biobased ethanol. In addition, microbial production of EG from plant-derived sugars, i.e., d-glucose and d-xylose, has been extensively studied by several groups (Salusjärvi et al. 2019). Recent development of electrochemical and chemical processes enabled selective production of EG from CO_2_ via ethylene (Tamura et al. 2015; Lum et al. 2020; Leow et al. 2020; Fan et al. 2023). These technological advances expanded the potential role of EG in addition to traditional applications; EG is now becoming a promising feedstock that can be obtained from CO_2_. Furthermore, EG also can be obtained from syngas (Satapathy et al. 2018; Sun and Chai 2022), lignocellulolytic biomass (Pang et al. 2011; Li et al. 2012; te Molder et al. 2021), and PET wastes (Werner et al. 2021; Diao et al. 2023); all of them do not compete with human food stocks (Fig. 1). In this context, microbial conversion of EG to value-added chemicals is gaining attention as a sustainable process (Wagner et al. 2023b) (Fig. 1). In order to design and construct microbial cell factories with EG as the starting material, microbes capable of assimilating EG are useful as chassis for synthetic pathways converting EG to value-added chemicals.

**Fig. 2:**
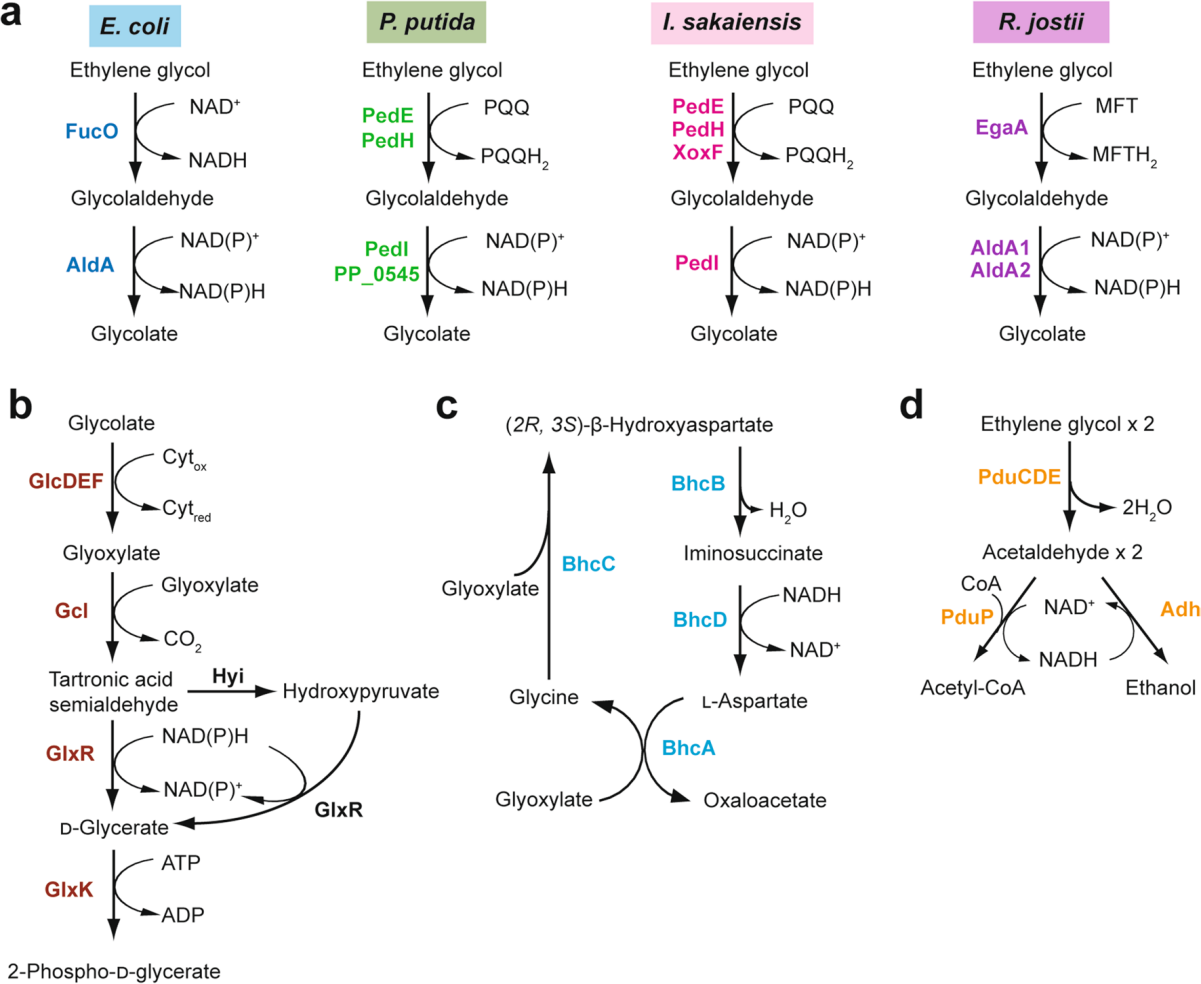
Ethylene glycol catabolism in microbes. **a** Sequential oxidation of ethylene glycol to glycolate in *E. coli*, *P. putida*, *I. sakaiensis*, and *R. jostii*. **b** The glycerate pathway and **c** the β-hydroxyaspartate cycle for glyoxylate assimilation. **d** The anaerobic ethylene glycol catabolism in *A. woodii*

EG assimilation has been reported in diverse microbes including *Clostridium glycolicum* (Gaston and Stadtman 1963), *Flavobacterium* sp. (Child and Willetts 1978), *Halomonas elongate* (Gonzalez et al. 1972), *Escherichia coli* (Boronat et al. 1983), *Pseudomonas putida* (Mückschel et al. 2012), *Acetobacterium woodii* (Trifunović et al. 2016), *Ideonella sakaiensis* (Hachisuka et al. 2022), *Rhodococcus jostii* (Shimizu et al. 2024), and *Paracoccus denitrificans* (Bordel et al. 2024). Microbial EG catabolism exhibits diversity in routes and enzymes involved in Fig. 2. The aerobic EG catabolism proceeds via a common sequential oxidation of EG to glyoxylate, where enzymes and cofactors involved in the initial oxidation of EG are varied among microbes (Fig. 2a). Subsequently, the resulting glyoxylate is further assimilated via the glycerate pathway (Fig. 2b) or the β-hydroxyaspartate cycle (BHAC) (Fig. 2c). In the anaerobic EG catabolism found in acetogens such as *A. woodie*, EG is dehydrated to acetaldehyde by diol dehydratase (Fig. 2d). In this mini-review, we describe EG catabolic pathways and catabolic enzymes in several microbes, and further review their applications for microbial conversion of EG to value-added chemicals including strain improvement by metabolic engineering.

## FucO-mediated ethylene glycol catabolism in *Escherichia coli*

*E. coli* is a gram-negative model bacterium providing a number of genetic tools for design and strain construction for the bioproduction of a variety of chemicals from renewable resources. Although the wild-type *E. coli* is unable to use EG, spontaneous mutants obtained from l-1,2-propanediol utilizing mutants were able to grow with EG as a sole source of carbon and energy (Boronat et al. 1983). It has been proposed that constitutive expression of *fucO* encoding 1,2-propandiol oxidoreductase and *aldA* encoding glycolaldehyde dehydrogenase is essential for EG catabolism by *E. coli*, where FucO catalyzes NAD^+^-dependent oxidation of EG to glycolaldehyde (Boronat and Aguilar 1979), and AldA catalyzes NAD(P)^+^-dependent oxidation of glycolaldehyde to glycolate (Caballero et al. 1983) (Fig. 2a). It should be noted that a recent study revealed that expression of *fucO* and deletion of *yqhD* encoding highly active aldehyde reductase is sufficient for EG assimilation by *E. coli* (Frazão et al. 2023). After the reactions catalyzed by FucO and AldA, the resulting glycolate is oxidized by glycolate dehydrogenase (GlcDEF) to glyoxylate (Pellicer et al. 1996), and further catabolized to 2-phospho-d-glycerate, an intermediate of glycolysis, via the glycerate pathway (Fig. 2b) including glyoxylate carboligase, (Gcl) (Chang et al. 1993), tartronate semialdehyde reductase (GlxR) (Njau et al. 2000), and d-glycerate 2-kinase (GlxK) (Rodionova et al. 2023). Note that GlxK was initially reported to phosphorylate the C3-position of d-glycerate (Doughty et al. 1966), whereas subsequent studies revealed that GlxK actually phosphorylates the C2-position of d-glycerate (Bartsch et al. 2008; Zelcbuch et al. 2015).

FucO, the key enzyme of EG catabolism in *E. coli*, is a member of group III alcohol dehydrogenase, which contains Fe(II) atom at the catalytic center (Montella et al. 2005; Zavarise et al. 2023). FucO is inactivated under aerobic conditions and physiologically catalyzes the reduction of l-lactaldehyde to l-1,2-propanediol for oxidation of NADH during anaerobic l-fucose and l-rhamnose utilization (Chen et al. 1987). The oxygen sensitivity of FucO is important to prevent the formation of l-1,2-propanediol under aerobic conditions (Baldomà and Aguilar 1988), whereas I6L/L7V mutant of FucO is known to be oxygen-resistant (Lu et al. 1998).

Microbial conversion of EG to value-added chemicals by genetically engineered *E. coli* has been reported by several groups for the production of multiple target compounds (Table 1). Pandit and co-workers reported glycolate production from EG using *E. coli* with overexpression of oxygen-tolerant *fucO* and *aldA* (Pandit et al. 2021). Using the orthogonal matrix approach, they evaluated several feedstocks including glucose, xylose, formate, and EG as substrates for glycolate production and found that EG is the best substrate for glycolate production in *E. coli*. Subsequent flux-balance analysis and flux variability analysis revealed that the oxygen supply is important to control glycolate production by *E. coli*. Finally, the engineered *E. coli* strain produced a maximum titer of 10.4 g/L glycolate from EG under optimized conditions (Pandit et al. 2021). In this context, some yeast and acetic acid bacteria are also known to produce glycolate from EG without assimilation (Kataoka et al. 2001; Wei et al. 2009). The best example of glycolate production from EG is the cell reaction of the acetic acid bacterium *Gluconobacter oxydans*, which produced 63.3 g/L glycolate at a yield of 97.2% under optimized conditions (Hua et al. 2018).

**Table 1:** Summary of microbial conversion of ethylene glycol or its derivatives to value-added chemicals

Strain	Input	Product	Titer	Reference
*Escherichia coli* (pEG03*)	Ethylene glycol	Glycolic acid	10.4 g/L	Pandit et al. (2021)
*Pichia naganishii* AKU4267	Ethylene glycol	Glycolic acid	35.3 g/L	Kataoka et al. (2001)
*Gluconobacter oxydans* NL71	Ethylene glycol	Glycolic acid	63.3 g/L	Hua et al. (2018)
*Escherichia coli* EGT01	Ethylene glycol	l-Tyrosine	2.0 g/L	Panda et al. (2023)
*Escherichia coli* EGP01	Ethylene glycol	l-Phenylalanine	1.5 g/L	Panda et al. (2023)
*Escherichia coli* EGC02	Ethylene glycol	*p*-Coumaric acid	1.0 g/L	Panda et al. (2023)
*Escherichia coli* TW1356	Glycolaldehyde	2,4-Dihydroxybutyric acid	1.0 g/L	Frazão et al. (2023)
*Escherichia coli* TW1828	Ethylene glycol	2,4-Dihydroxybutyric acid	0.8 g/L	Frazão et al. (2023)
*Pseudomonas putida* MFL185	Ethylene glycol	Poly(hydroxyalkanoate)	0.32 g/g DCW	Franden et al. (2018)
*Pseudomonas putida* AW165	BHET	β-Ketoadipic acid	15.1 g/L	Werner et al. (2021)
*Pseudomonas putida* AW165	Depolymerized PET	β-Ketoadipic acid	0.22 g/L	Werner et al. (2021)
*Pseudomonas putida* Pp-TEP	PET hydrolysate	Poly(hydroxyalkanoate)	0.39 g/L	Bao et al. (2023)
*Pseudomonas putida* strains	PET hydrolysate	Poly(hydroxyalkanoate)	0.64 g/L	Bao et al. (2023)
*Pseudomonas putida* Pp-TEM	PET hydrolysate	*cis, cis*-Muconic acid	1.66 g/L	Bao et al. (2023)
*Pseudomonas putida* strains	PET hydrolysate	*cis, cis*-Muconic acid	4.73 g/L	Bao et al. (2023)
*Pseudomonas umsongensis* GO16	PET hydrolysate	Hydroxyalkanoyloxy-alkanoates	35 mg/L	Tiso et al. (2021)
*Rhodococcus jostii* PET	PET hydrolysate	Lycopene	1.3 mg/L	Diao et al. (2023)
*Ideonella sakaiensis* 201-F6	PET	Poly(hydroxyalkanoate)	0.75 g/L	Fujiwara et al. (﻿^2021^)

**Fig. 3:**
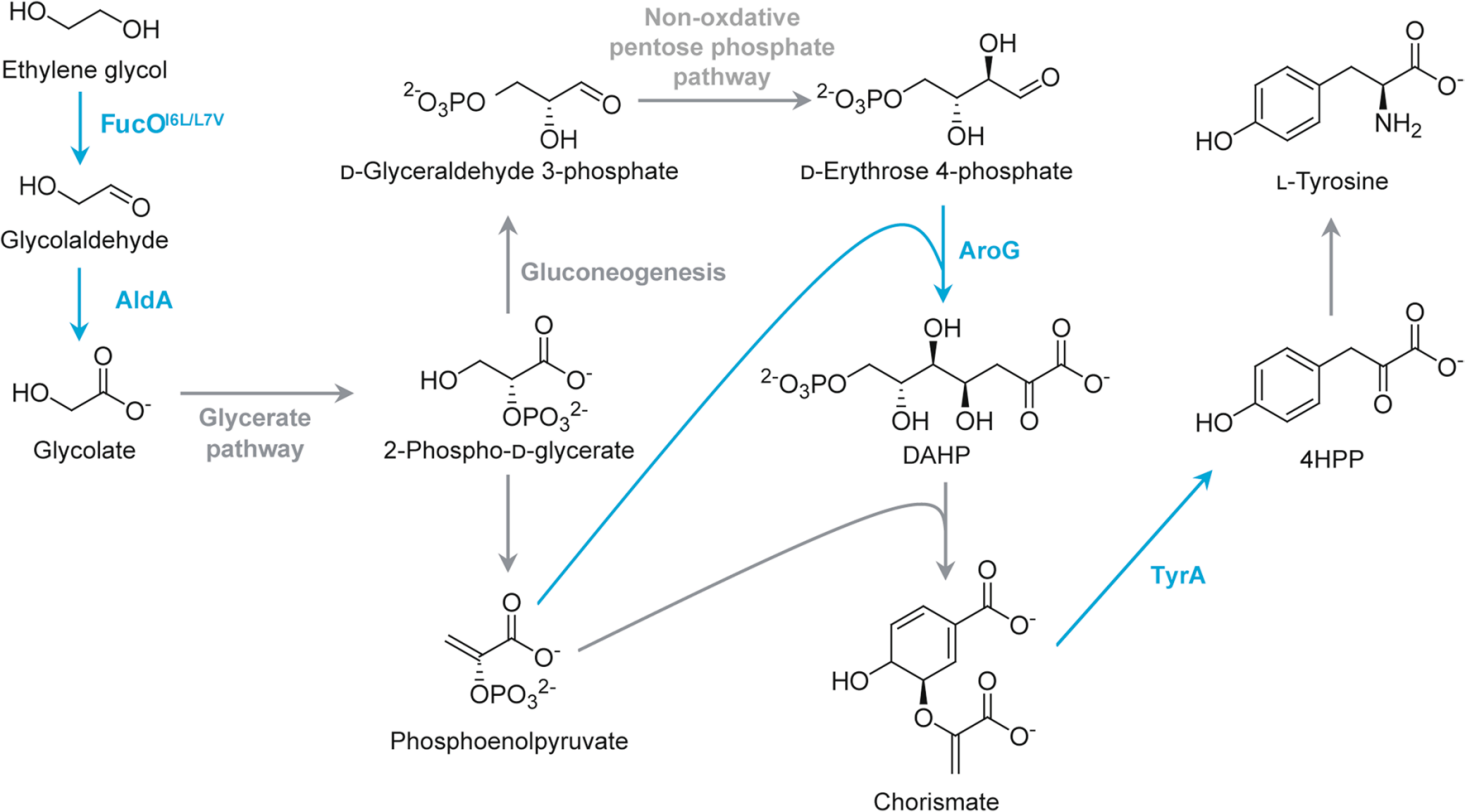
Microbial conversion of ethylene glycol to l-tyrosine by genetically engineered *E. coli*. Overexpressed genes are shown in blue. FucO^I6L/L7V^, I6L/L7V mutant of l-1,2-propandiol oxidoreductase; AldA, l-lactaldehyde dehydrogenase; AroG, DAHP synthase; TyrA, chorismate mutase/prephenate dehydrogenase; DAHP, 3-deoxy-d-arabino-heptulosonate 7-phosphate; 4HPP, 4-hydroxyphenylpyruvate

Panda and co-workers recently reported the conversion of EG to aromatic compounds including l-tyrosine, l-phenylalanine, and *p*-coumarate using genetically engineered *E. coli* (Table 1) with plasmids expressing oxygen-tolerant *fucO*, *aldA*, feedback-resistant *tyrA*, and feedback-resistant *aroG* (Panda et al. 2023) (Fig. 3). The best strain produced 2 g/L l-tyrosine from 10 g/L EG, which corresponds to almost 50% of theoretical yield. A similar titer was achieved when acid hydrolysates of PET waste were used as the starting material.

**Fig. 4:**
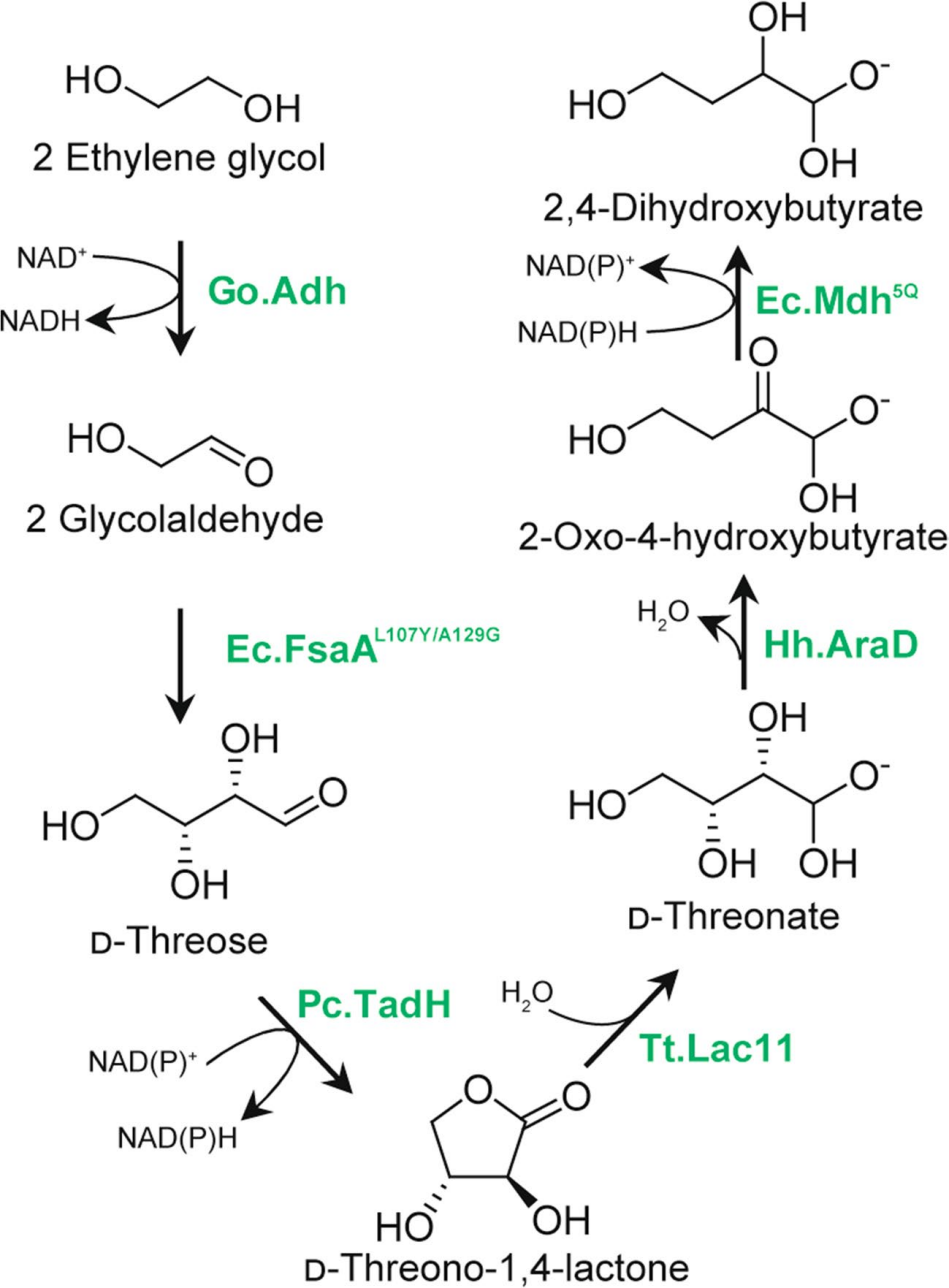
Microbial conversion of ethylene glycol to 2,4-dihydroxybutyrate by genetically engineered *E. coli*. GO.Adh, NAD^+^-dependent alcohol dehydrogenase Gox0313 from *G. oxydans*; Ec.FsaA^L107Y/A129G^, L107Y/A129G mutant of d-fructose 6-phosphate aldolase from *E. coli*; Pc.TadH, d-threo-aldose 1-dehydrogenase from *Paraburkholderia caryophylli*; Tt.Lac11, gluconolactonase from *Thermogutta terrifontis*; Hh.AraD, d-arabinonate dehydratase from *Herbaspirillum huttiense*; Ec.Mdh^5Q^, I12V/R81A/M85Q/D86S/G179D mutant of l-malate dehydrogenase from *E. coli*

Frazao and co-workers achieved the microbial production of 2,4-dihydroxybutyric acid (DHB), a non-natural precursor for a methionine analogue or 1,3-propanediol, from EG using *E. coli* implemented with a synthetic pathway (Frazão et al. 2023) (Fig. 4). They screened enzymes for the synthetic pathway for the conversion of glycolaldehyde to DHB. After establishment of an *E. coli* strain producing DHB from glycolaldehyde, the pathway was extended for EG utilization by introducing EG-oxidizing enzymes (Fig. 4). Notably, the best DHB titer from EG, 0.8 g/L (Table 1), was achieved by the expression of Gox0313 (Zhang et al. 2015), NAD^+^-dependent alcohol dehydrogenase from *G. oxydans*, not endogenous *fucO* nor its oxygen-tolerant mutant.

## PedEH-mediated ethylene glycol catabolism in *Pseudomonas putida*

*P. putida* is a gram-negative metabolically versatile bacterium that has been used for various biotechnological applications (Salusjärvi et al. 2019). Mückschel and co-workers examined the utilization of EG by two *P. putida* strains, JM37 and KT2440, and they found that *P. putida* JM37 was able to grow with EG as a sole source of carbon and energy, whereas *P. putida* KT2440 was unable to grow with EG (Mückschel et al. 2012). Proteome analysis revealed that PQQ-dependent alcohol dehydrogenases (PedEH), aldehyde dehydrogenase (PedI), and isocitrate lyase (AceA) were upregulated in *P. putida* KT2440 upon EG treatment, whereas Gcl was not (Mückschel et al. 2012). Subsequent adaptive laboratory evolution experiments revealed that the activation of the glycerate pathway including glyoxylate carboligase (Gcl), hydroxypyruvate isomerase (Hyi), tartronate semialdehyde reductase (GlxR), d-glycerate 2-kinase (TtuD), and pyruvate kinase (PyrK) by deletion of *gclR* encoding a GntR family transcriptional regulator enabled *P. putida* KT2440 to grow with EG as a sole source of carbon and energy (Li et al. 2019).

**Fig. 5:**
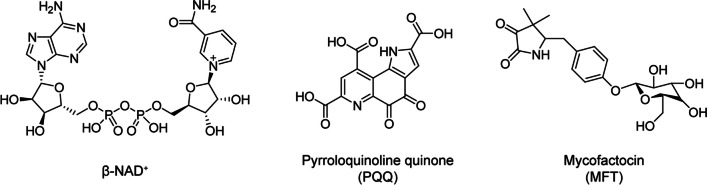
Chemical structure of cofactors involved in the oxidation of ethylene glycol. Chemical structures of electron acceptors involved in microbial oxidation of ethylene glycol are shown

A major difference between EG assimilation in *E. coli* and that in *P. putida* is enzymes involved in the oxidation of EG; FucO in *E. coli* uses NAD^+^ for the electron acceptor, whereas both PedE and PedH in *P. putida* use PQQ for the electron acceptor. As shown in Fig. 5, the chemical structures of NAD^+^ and PQQ are completely different. Unlike the ubiquitous molecule NAD^+^ found in all domains of life, PQQ is only found in some prokaryotes including methylotrophs (Keltjens et al. 2014), and that at least six genes are required for PQQ biosynthesis (Puehringer et al. 2008). A unique feature of PQQ is its high midpoint potential (90 mV), the value of which is significantly higher than that of NAD^+^ (− 340 mV). Notably, the high midpoint potential of PQQ is advantageous to drive EG oxidation by PedEH, as compared to FucO that rather prefers NADH-dependent reduction of glycolaldehyde at neutral pH (Boronat and Aguilar 1979).

**Fig. 6:**
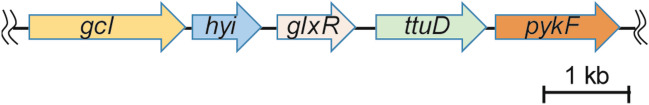
The *gcl* operon in *Pseudomonas putida* KT2440. *gcl*, glyoxylate carboligase; *hyi*, hydroxypyruvate isomerase; *glxR*, tartronate semialdehyde reductase; *ttuD*, d-glycerate 2-kinase; *pykF*, pyruvate kinase

Biotechnological applications of *P. putida* strains for microbial conversion of EG or its derivatives to value-added chemicals have been reported by several groups (Table 1). The earlier work is poly(hydroxyalkanoate) (PHA) production from EG reported by Franden and co-workers (Franden et al. 2018). They firstly analyzed the minimum genes in the *gcl* operon required for EG assimilation by *P. putida*. Among genes in the *gcl* operon (Fig. 6), only two genes, *gcl* and *glxR*, were required for growth with EG, whereas expression of the entire *gcl* operon further improved growth with EG. It was also revealed that overexpression of *glcDEF* along with overexpression of the glycerate pathway improved EG utilization by *P. putida*. The resulting strain MFL185 was able to consume 500 mM EG within 120 h, and produced 32.19% dry cell weight of C8-C14 PHA from EG (Table 1).

**Fig. 7:**
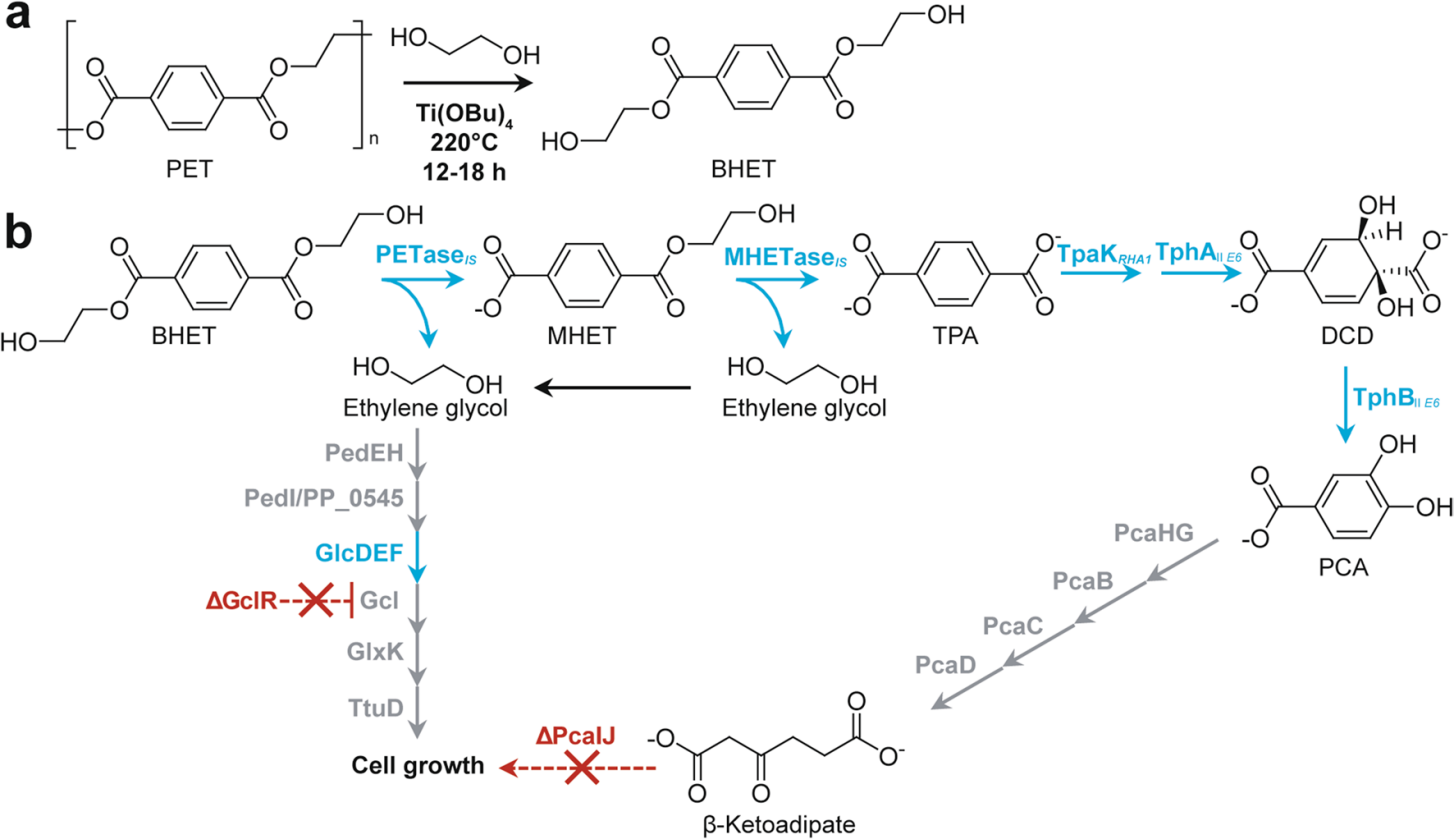
Chemoenzymatic synthesis of β-ketoadipate from poly(ethylene terephthalate). **a** Chemical depolymerization of poly(ethylene terephthalate) (PET) to bis(2-hydroxyethyl)terephthalate (BHET) catalyzed by titanium butoxide in ethylene glycol. **b** Microbial conversion of BHET to β-ketoadipate by genetically engineered *P. putida*. Overexpressed genes and deleted genes are shown in blue and red, respectively. PETase_*IS*_, PET hydrolase from *I. sakaiensis*; MHETase_*IS*_, mono(2-hydroxyethyl) terephthalate (MHET) hydrolase from *I. sakaiensis*; TpaK_*RHA1*_, terephthalate (TPA) transporter from *R. jostii* RHA1; TphA_II *E6*_, terephthalate 1,2-dioxygenase from *Comamonas* sp. E6; TphB_II *E6*_, 1,2-dihydroxy-3,5-cyclohexadiene-1,4-dicarboxylate (DCD) dehydrogenase from *Comamonas* sp. E6; GlcDEF, endogenous glycolate dehydrogenase

Werner and co-workers achieved β-ketoadipic acid (βKA) production from bis(2-hydroxyethyl)terephthalate (BHET), 1.5-mer of PET, or chemically depolymerized PET using genetically engineered *P. putida* (Werner et al. 2021). Based on EG-assimilating strain Δ*gclR*, deletion of *pcaIJ* involved in βKA assimilation (Parales and Harwood 1992, 1993) and introduction of terephthalate (TPA) catabolic genes (*tphA2*_*II*_*A3*_*II*_*B*_*II*_*A1*_*II*_) from *Comamonas* sp. E6 (Sasoh et al. 2006), TPA transporter (*tpaK*) from *R. jostii* RHA1 (Hara et al. 2007), and PET-degrading genes from *I. sakaiensis* (Yoshida et al. 2016) resulted in direct conversion of BHET or depolymerized PET to βKA (Fig. 7) with titers of 15.1 g/L and 0.22 g/L, respectively (Werner et al. 2021) (Table 1). Notably, accumulation of EG was observed during βKA production from BHET, suggesting that βKA inhibited EG utilization of *P. putida* by an unknown mechanism (Werner et al. 2021). In order to utilize both TPA and EG efficiently, Bao and co-workers used the consortium involving two *P. putida* strains, specializing in TPA and EG utilization, respectively (Bao et al. 2023). Comparing to the mono-culture approach, the consortium produced higher titer of PHA (0.64 g/L) and *cis*, *cis*-muconic acid (4.73 g/L) from PET hydrolysate, respectively (Table 1).

Tiso and co-workers investigated hydroxyalkanoyloxy-alkanoates (HAA) production from enzymatically depolymerized PET (Tiso et al. 2021) by using *Pseudomonas umsongensis* GO16 capable of assimilating TPA with endogenous TPA catabolic genes (Narancic et al. 2021). Since the strain GO16 was unable to utilize EG, they performed adaptive laboratory evolution. As a result, the obtained EG-assimilating mutant strain GO16 KS3 produced 35 mg/L HAA from enzymatically depolymerized PET (Table 1). Nevertheless, HAA production by the strain KS3 solely relied on TPA (Tiso et al. 2021).

## EgaA-mediated ethylene glycol catabolism in *Rhodococcus jostii*

*R. jostii* is a gram-positive mycolic acid-containing bacterium known to be able to degrade various aromatic compounds including TPA (Hara et al. 2007). Due to its metabolic versatility and availability of tools for genome engineering (Liang and Yu 2021; Round et al. 2021), *R. jostii* has been used for several biotechnological applications (Donini et al. 2021). EG utilization by *R. jostii* was initially reported by Diao and co-workers in an attempt for upcycling of PET waste to lycopene by engineered strains derived from *R. jostii* PET (Diao et al. 2023); however, the EG catabolic pathway and EG catabolic enzymes in *R. jostii* PET were yet to be elucidated. Recently, we found that *R. jostii* strain RHA1 also can grow with EG as a source of carbon and energy (Shimizu et al. 2024). Biochemical and genetic analyses revealed that a mycofactocin (MFT)-associated dehydrogenase (EgaA) is responsible for the oxidation of EG to glycolaldehyde, and two aldehyde dehydrogenases (AldA1 and AldA2) might be involved in the oxidation of glycolaldehyde to glycolate, which is further catabolized by the glycerate pathway (Shimizu et al. 2024). It has been also reported that RHA1_ro02984 protein catalyzes consecutive oxidation of glycolaldehyde to oxalate during lignin oxidation by *R. jostii* RHA1 (Alruwaili et al. 2023), whereas the corresponding gene was not upregulated during growth with EG (Shimizu et al. 2024).

A unique feature of EG catabolism in *R. jostii* is the involvement of a MFT-associated dehydrogenase in the oxidation of EG (Fig. 2a). MFT is a group of ribosomally synthesized and posttranslationally modified peptides (Fig. 5) that has been suggested to act as a redox cofactor for the oxidation of various alcohols in Actinobacteria including the genus *Rhodococcus* (Ayikpoe et al. 2019). Indeed, the deletion of *egaA* negatively affected ethanol, 1-propanol, l-1,2-propanediol, and 1-butanol assimilation in addition to EG, suggesting that EgaA is responsible for the oxidation of various alcohols in *R. jostii* RHA1 (Shimizu et al. 2024). Although the midpoint potential of MFT is currently not known, that of premycofactocin, a precursor of MFT lacking the glycosyl-moiety, has been reported to be − 225 mV (Ayikpoe and Latham 2019).

As *R. jostii* is able to assimilate both EG and TPA constituting PET, this bacterium is a promising chassis for upcycling of PET to value-added chemicals. In this context, Diao and co-workers examined the upcycling of alkaline hydrolysates of PET to lycopene using genetically engineered *R. jostii* PET (Diao et al. 2023). The study revealed that the bacterium can use EG and TPA simultaneously, and achieved approximately 1300 µg/L lycopene from alkaline hydrolysates of PET by deletion of a putative lycopene β-cyclase gene (*crtL*-b) and optimization of the 2-methylerythritol 4-phosphate pathway for carotenoid biosynthesis (Diao et al. 2023).

## Ethylene glycol catabolism in the PET assimilating bacterium *Ideonella sakaiensis*

*I. sakaiensis* is a gram-negative bacterium capable of assimilating PET as a sole source of carbon and energy (Yoshida et al. 2016). Since PET is a co-polymer consisting of EG and TPA, equivalent moles of EG and TPA are released during PET degradation catalyzed by PETase (Liu et al. 2023) and MHETase (Palm et al. 2019) produced by *I. sakaiensis*. Based on genome information, *I. sakaiensis* was predicted to be able to utilize TPA (Yoshida et al. 2016), and a recent study revealed that *I. sakaiensis* is able to grow with EG as a sole source of carbon and energy (Hachisuka et al. 2022). Genetic and biochemical analyses revealed that *I. sakaiensis* uses three PQQ-dependent alcohol dehydrogenases, PedE, PedH, and XoxF, for the oxidation of EG to glycolaldehyde and aldehyde dehydrogenase (PedI) for NAD^+^-dependent oxidation of glycolaldehyde to glycolate (Hachisuka et al. 2022) (Fig. 2a). Among three PQQ-dependent alcohol dehydrogenases, PedE exhibited Ca^2+^-dependent dehydrogenase activity towards various alcohols, whereas PedH and XoxF exhibited Pr^3+^-dependent dehydrogenase activities, where PedH preferred short-chain alcohols and XoxF preferred long-chain alcohols (Hachisuka et al. 2022). Based on genome information, *I. sakaiensis* was proposed to use the glycerate pathway for glyoxylate assimilation.

Since *I. sakaiensis* can utilize PET directly without chemical depolymerization, the application of this bacterium enables one-pot bioconversion of PET to value-added chemicals. The pioneering work by Fujiwara and co-workers is upcycling of PET to PHA using the wild-type *I. sakaiensis* (Fujiwara et al. 2021). They found PHA biosynthetic genes in the genome of *I. sakaiensis*, and confirmed PHA accumulation during growth with PET. After optimization of culture conditions, *I. sakaiensis* accumulated PHA from PET up to 48% of dry cell weight, which corresponds to PHA titer of 0.75 g/L (Table 1). In addition, a subsequent study revealed substrate specificity of PHA synthase from *I. sakaiensis* (Tan et al. 2022). Recently, a protocol for gene manipulation of *I. sakaiensis* was established (Hachisuka et al. 2021), enabling metabolic engineering of *I. sakaiensis* for the production of various chemicals from PET waste.

## Ethylene glycol assimilation via the β-hydroxyaspartate cycle in *Paracoccus denitrificans*

*P. denitrificans* is a gram-negative bacterium that has been known to assimilate glyoxylate via the BHAC (Kornberg and Morris 1965). Recent study elucidated four enzymes constituting the BHAC (Schada von Borzyskowski et al. 2019): aspartate-glyoxylate aminotransferase (BhcA), β-hydroxyaspartate dehydratase (BhcB), β-hydroxyaspartate aldolase (BhcC), and iminosuccinate reductase (BhcD) (Fig. 2c). This study also revealed that the BHAC is distributed in a certain population of marine bacteria to use glycolate produced by marine algae and seaweeds via the oxygenase reaction of rubisco (Schada von Borzyskowski et al. 2019). Very recently, Bordel and co-workers found that *P. denitrificans* is able to grow with EG as a source of carbon and energy (Bordel et al. 2024). Based on its genome information, *P. denitrificans* is predicted to use FucO, AldA, and GlcDEF for sequential oxidation of EG to glyoxylate, which is further converted to oxaloacetate via the BHAC for biomass and energy (Bordel et al. 2024).

The BHAC is considered to be a more efficient carbon conserving C2-assimilating pathway than the glycerate pathway since the BHAC does not produce CO_2_ and does not require ATP for glyoxylate assimilation (Schada von Borzyskowski et al. 2019; Borzyskowski et al. 2020; Diehl et al. 2023) (Fig. 2c). Indeed, the implementation of the BHAC to the *gcl* deletion mutant of *P. putida* KT2440 and adaptive laboratory evolution resulted in both higher growth rates and biomass yields with EG as compared to the E6.1 strain that has been evolved to assimilate EG by the glycerate pathway (von Borzyskowski et al. 2023).

## Anaerobic ethylene glycol catabolism in *Acetobacterium woodii*

The obligate anaerobic acetogenic bacterium *A. woodie* can grow with EG under strict anaerobic conditions with the formation of acetate and ethanol (Trifunović et al. 2016). Although aerobic EG catabolic pathways start with the oxidation of EG to form glycolaldehyde, the anaerobic EG catabolic pathway in *A. woodii* starts with the dehydration of EG to form acetaldehyde by propanediol dehydratase (PduCDE), which is known to be very oxygen-sensitive (Hartmanis and Stadtman 1986). The resulting acetaldehyde is further oxidized by CoA-dependent propionaldehyde dehydrogenase (PduP) to form acetyl-CoA with concomitant reduction of NAD^+^ to NADH, which is assumed to be re-oxidized to NAD^+^ through the reduction of acetaldehyde to ethanol by a yet-to-be identified alcohol dehydrogenase (Trifunović et al. 2016) (Fig. 2d). Notably, PduCDEP are also responsible for l-1,2-propanediol catabolism by *A. woodii*, which involves the formation of bacterial microcompartments presumably for the protection of cells from toxic aldehyde intermediates (Schuchmann et al. 2015).

In the purpose of bioconversion of EG to value-added chemicals, the use of acetogenic bacteria including *A. woodii* would be challenging since they obligately accumulate acetate and/or alcohols during growth to gain ATP and mediate cellular redox-state under anaerobic conditions (Katsyv and Müller 2020). However, it should be noted that acetogenic bacteria are capable of utilizing CO_2_ as the carbon source via the Wood-Ljungdahl pathway (Basen and Müller 2023), allowing direct bioconversion of CO_2_ to value-added chemicals including EG (Liew et al. 2022; Bourgade et al. 2022).

## Conclusions and future prospects

As reviewed here, minimum requirements for the aerobic EG utilization are likely to be two metabolic modules: (i) oxidative conversion of EG to glyoxylate (three reactions) and (ii) glyoxylate assimilation via either the glycerate pathway (three reactions) or the BHAC (four reactions). Therefore, conferring the ability for EG utilization to non-EG-assimilating microbes would be time-consuming since enzymes for at least six reactions are required to be implemented. It should be noted that one step may require multiple genes such as for cofactor biosynthesis. In this regard, EG-utilizing microbes are useful as chassis for microbial cell factories from EG as the starting material since they do not require metabolic engineering for EG utilization.

Although EG catabolic pathways in diverse microbes have been characterized so far, their regulation seems to be less studied. For microbial conversion of EG to value-added chemicals, target compounds and/or metabolic intermediates might affect the regulation of EG catabolic enzymes unexpectedly as observed in the inhibition of EG utilization during β-KA production by *P. putida* (Werner et al. 2021). Thus, understanding the regulation of EG catabolic enzymes at both the transcriptional level and the post-translational level including allosteric regulation, substrate inhibition, product inhibition, and feedback inhibition would be required to improve the performance of microbial cell factories with EG and/or PET wastes as the starting materials.

**Fig. 8:**
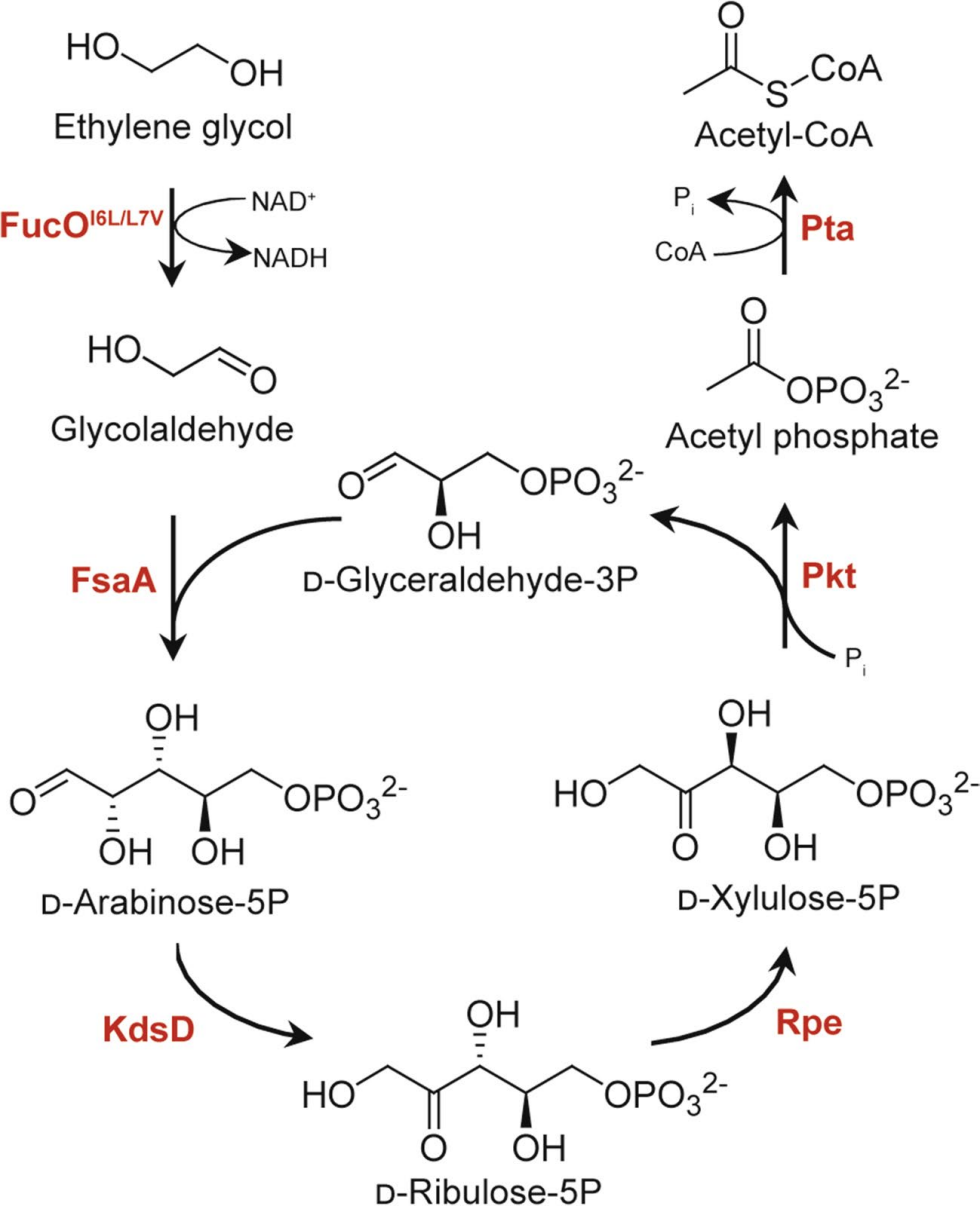
A non-natural pathway for carbon conserving conversion of ethylene glycol to acetyl-CoA. FucO^I6L/L7V^, I6L/L7V mutant of L-1,2-propandiol oxidoreductase from *E. coli*; FsaA, d-fructose 6-phosphate aldolase from *E. coli*; KdsD, d-arabinose 5-phosphate isomerase from *E. coli*; Rpe, d-ribulose 5-phosphate epimerase from *E. coli*; Pkt, phosphoketolase from *Clostridium acetobutylicum*; Pta, phosphate acetyltransferase from *E. coli*

In addition to natural EG-assimilating pathways, non-natural synthetic pathways for glycolaldehyde assimilation have been designed and validated for in vitro functionality (Yang et al. 2019; Mao et al. 2021; Scheffen et al. 2021). Notably, Wagner and co-workers demonstrated in vivo functionality of a synthetic pathway for the conversion of EG to acetyl-CoA in *E. coli* (Fig. 8) albeit at currently extremely small yields (Wagner et al. 2023a). Further design and improvement of non-natural pathways for EG utilization and a combination of those with natural pathways would be a future direction.

Although microbial processes are expected to reduce energy costs and CO_2_ emission as compared to conventional chemical processes, most microbial processes have been designed to produce bio-chemicals or bio-fuels from edible biomass such as glucose, which would ultimately compete with food stocks (Dwi Prasetyo et al. 2020). In this regard, EG is a promising next-generation feedstock that can be derived from materials not competing food stocks, and EG preparation by means of sustainable process is rapidly developing. Thus, research on microbial conversion of EG to value-added chemicals will contribute to the development of a sustainable industry in parallel with research on microbial conversion of other potential next-generation feedstocks such as syngas (Kim et al. 2023), CO_2_ (Nisar et al. 2021), methanol (Zhang et al. 2019), and acetate (Kiefer et al. 2021).
